# Exposure to Pesticides and Health Effects on Farm Owners and Workers From Conventional and Organic Agricultural Farms in Costa Rica: Protocol for a Cross-Sectional Study

**DOI:** 10.2196/10914

**Published:** 2019-01-25

**Authors:** Samuel Fuhrimann, Mirko S Winkler, Philipp Staudacher, Frederik T Weiss, Christian Stamm, Rik IL Eggen, Christian H Lindh, José A Menezes-Filho, Joseph M Baker, Fernando Ramírez-Muñoz, Randall Gutiérrez-Vargas, Ana M Mora

**Affiliations:** 1 School of Public Health and Family Medicine Faculty of Health Sciences University of Cape Town Cape Town South Africa; 2 Swiss Tropical and Public Health Institute Basel Switzerland; 3 University of Basel Basel Switzerland; 4 Swiss Federal Institute of Aquatic Science and Technology (Eawag) Dübendorf Switzerland; 5 Institute of Biogeochemistry and Pollutant Dynamics Department of Environmental Systems Science ETH Zürich Zürich Switzerland; 6 Division of Occupational and Environmental Medicine Institute of Laboratory Medicine Lund University Lund Sweden; 7 Laboratory of Toxicology Faculty of Pharmacy Federal University of Bahia Bahia Brazil; 8 Department of Psychiatry and Behavioral Sciences School of Medicine Stanford University Stanford, CA United States; 9 Central American Institute for Studies on Toxic Substances Universidad Nacional Heredia Costa Rica; 10 Centro de Investigación y Diagnóstico en Salud y Deporte Universidad Nacional Heredia Costa Rica

**Keywords:** acetylcholinesterase, agriculture, Costa Rica, farm workers, near-infrared spectroscopy, neurobehavioral outcomes, pesticides, pesticide exposure assessment, respiratory outcomes

## Abstract

**Background:**

Pesticide use is increasing in low- and middle-income countries (LMICs) including Costa Rica. This increase poses health risks to farm owners, farm workers, and communities living near agricultural farms.

**Objective:**

We aimed to examine the health effects associated with occupational pesticide exposure in farm owners and workers from conventional and organic smallholder farms in Costa Rica.

**Methods:**

We conducted a cross-sectional study involving 300 owners and workers from organic and conventional horticultural smallholder farms in Zarcero County, Costa Rica. During the baseline study visit, we administered a structured, tablet-based questionnaire to collect data on sociodemographic characteristics, pesticide exposure, and health conditions (eg, respiratory and allergic outcomes and acute pesticide intoxication symptoms) and administered a neurobehavioral test battery (eg, Finger Tapping Test and Purdue Pegboard); we measured blood pressure, anthropometry (height, weight, and waist circumference), and erythrocytic acetylcholinesterase activity and also collected urine samples. In addition, a functional neuroimaging assessment using near-infrared spectroscopy was conducted with a subset of 50 study participants. During the follow-up study visit (~2-4 weeks after the baseline), we administered participants a short questionnaire on recent pesticide exposure and farming practices and collected hair, toenail, and urine samples. Urine samples will be analyzed for various pesticide metabolites, whereas toenails and hair will be analyzed for manganese (Mn), a biomarker of exposure to Mn-containing fungicides. Self-reported pesticide exposure data will be used to develop exposure intensity scores using an exposure algorithm. Furthermore, exposure-outcome associations will be examined using linear and logistic mixed-effects regression models.

**Results:**

Fieldwork for our study was conducted between May 2016 and August 2016. In total, 113 farm owners and 187 workers from 9 organic and 83 conventional horticultural smallholder farms were enrolled. Data analyses are ongoing and expected to be published between 2019 and 2020.

**Conclusions:**

This study is one of the first to examine differences in health effects due to pesticide exposure between farm owners and workers from organic and conventional smallholder farms in an LMIC. We expect that this study will provide critical data on farming practices, exposure pathways, and how occupational exposure to pesticides may affect farm owners and workers’ health. Finally, we hope that this study will allow us to identify strategies to reduce pesticide exposure in farm owners and workers and will potentially lay the groundwork for a future longitudinal study of health outcomes in farm owners and workers exposed to pesticides.

**International Registered Report Identifier (IRRID):**

DERR1-10.2196/10914

## Introduction

Pesticides are extensively used in agriculture and for control of vector-borne diseases across the globe [1,2]. Current data from the Food and Agriculture Organization suggest that pesticide use is increasing globally, with its largest growth in low- and middle-income countries (LMICs) in tropical contexts [3]. Notably, registered pesticides are often not assessed in tropical contexts, where decay rates of active ingredients and metabolites of pesticides differ from other settings [4] and regulatory bodies often fail to phase-out harmful pesticides or monitor their safe use [5]. In LMICs, the smallholder farming sector frequently struggles to use pesticides safely due to a lack of awareness and low risk perception among farm owners and workers [6-8].

Pesticide applicators (either farm owners or workers) in smallholder farms are often exposed to these chemicals at different stages of the process (eg, storage, mixing, preparation, and application) [9,10]. Therefore, uncontrolled and uninformed pesticide use can directly expose workers and surrounding communities through drift and pesticide residues in food and drinking water [11]. Several studies from LMICs have shown that acute pesticide poisoning represents an important cause of morbidity and mortality among farm workers [12]. In addition, long-term exposure to pesticides such as organophosphates and carbamates has been linked to a broad range of chronic health effects, including impaired neurobehavioral function (eg, cognitive and behavioral disorders), respiratory problems, obesity, and diabetes [13-17].

The characterization of pesticide exposures in LMICs is challenging due to the short half-lives of most of these chemicals in the human body, limited availability of biomarkers of exposure, and lack of epidemiological data [18-20]. As highlighted by a recent descriptive review [19], most studies in LMICs have relied on self-reported pesticide exposures. A few studies have generated pesticide exposure matrices, estimating exposure intensity indices using the amount of pesticide used and personal protective equipment worn during the applications [18,21,22]. However, these exposure matrices are prone to information bias and often lack validation against biomarkers of exposure in humans (eg, urine and blood) [18,19].

Several studies have examined the health effects of occupational pesticide exposure in tropical settings [23-29]. In addition, multiple studies outside of LMICs have assessed differences in pesticide use practices from conventional farming systems (ie, using synthetic pesticides) and organic (ie, using biological pest control; certified as organic by third-party agencies) farms [30]. Nevertheless, to the best of our knowledge, only one published study from Portugal has compared pesticide exposure and health outcomes in farm workers from both types of farms [31]. In the present study, we aimed to determine whether occupational pesticide exposure (assessed through self-reported data and biomarkers of exposure) affects the health of farm owners and workers from conventional and organic smallholder farms in Costa Rica.

## Methods

### Objectives and Study Design

**Figure 1:**
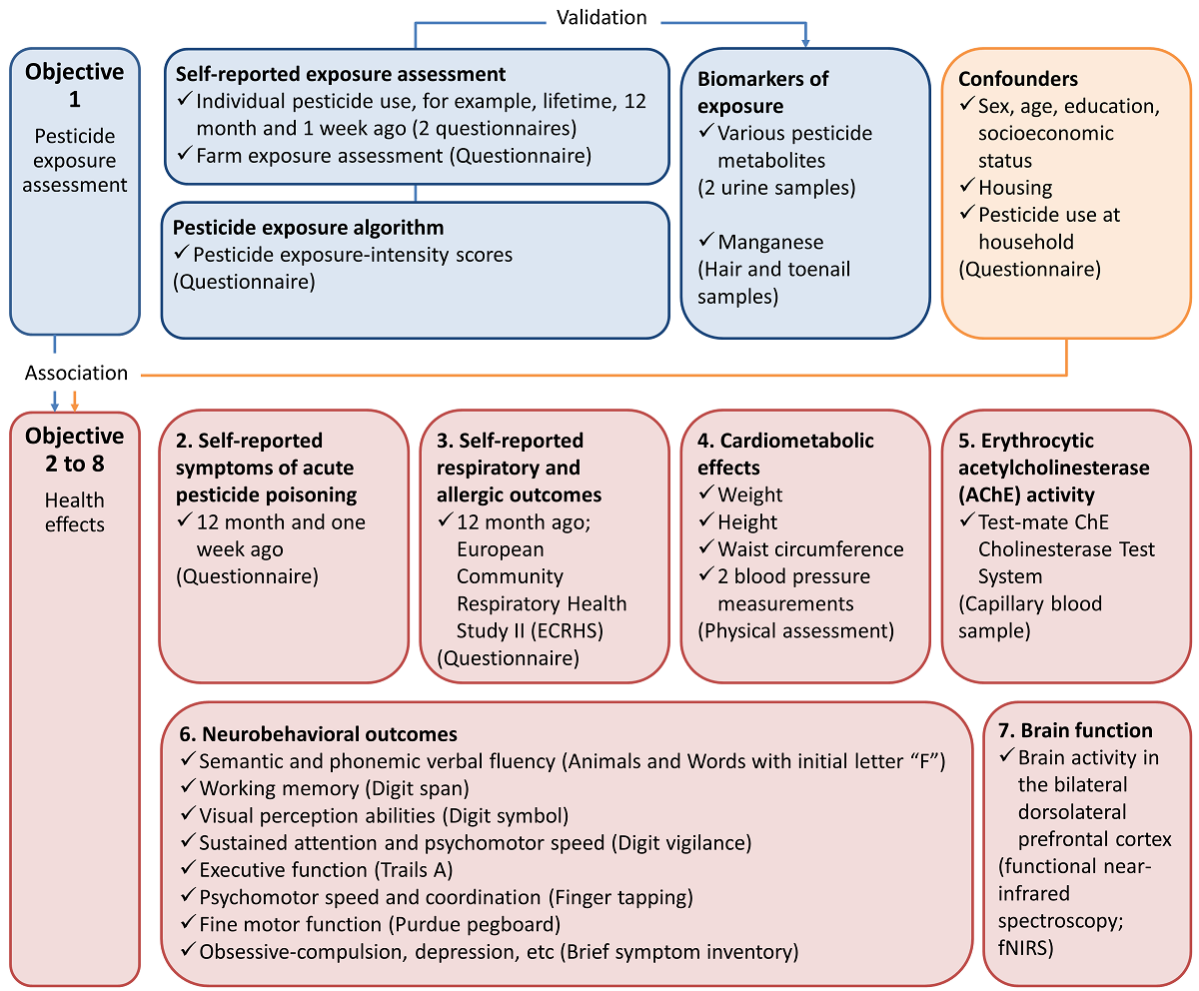
Aims (bold text), research design details and tools used (tick) in the study conducted in Zarcero County, Costa Rica, 2016.

We conducted a cross-sectional study of 300 farm owners and workers with repeated exposure assessment at two time points (to study the variability of pesticide exposure over time) between May 2016 and August 2016 (rainy season, during which the highest pesticide application is expected). The specific objectives of the project are as follows (Figure 1):

1. To assess occupational pesticide exposure in owners and workers of conventional and organic farms, using self-reported pesticide use data and biomarkers of pesticide exposure.
2. To evaluate the association of occupational pesticide exposure (determined using a pesticide exposure matrix and also biomarkers of exposure) with self-reported symptoms of acute intoxication in the last 12 months.
3. To examine the association of occupational pesticide exposure with self-reported respiratory and allergic outcomes in the last 12 months.
4. To evaluate the association between occupational pesticide exposure and cardiometabolic effects, such as adiposity and high blood pressure.
5. To assess the association of occupational exposure to organophosphates and carbamates with erythrocytic acetylcholinesterase (AChE) activity.
6. To assess the association of occupational pesticide exposure with neurobehavioral outcomes, such as working memory, visual perception, and fine motor function.
7. To examine the association of occupational pesticide exposure with changes in brain activity, assessed using functional near-infrared spectroscopy (fNIRS).

This study is part of the Pesticide Use in Tropical Settings (PESTROP) Project, which aims to deepen our understanding of the environmental, health, and regulatory dimensions of pesticide use in conventional and organic agriculture in LMICs. The design of our research was partly informed by a study conducted in the same study area between 2014 and 2016 [32]. This study focused on the diagnosis of pesticide use in farms with conventional practices and highlighted unintended uses of pesticides among farm owners in Zarcero (eg, use of pesticides without appropriate training and personal protective equipment protection and discharge of pesticide containers into the environment). The findings of this study provided orientation on exposure pathways to be expected and influenced the definition of our study groups.

**Figure 2:**
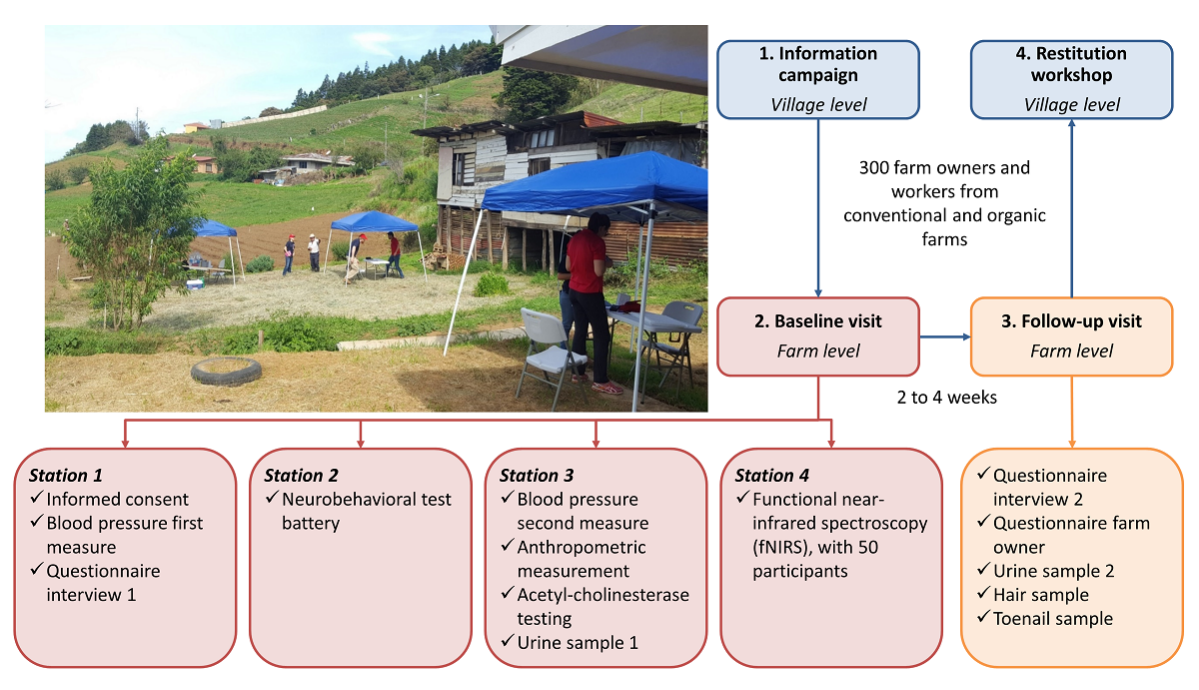
Diagram of the fieldwork setup in the Zarcero study, Costa Rica, 2016.

All study materials and procedures were approved by the human subjects committee of the Universidad Nacional in Costa Rica (UNA-CECUNA-ACUE-04-2016) and Ethical Board of the Ethikkommission Nordwest- und Zentralschweiz in Switzerland (EKNZ-UBE 2016-00771). Written informed consent was obtained from all study participants at enrollment. Study results will be communicated back to participants and stakeholders at restitution workshops (Figure 2).

### Study Area

**Figure 3:**
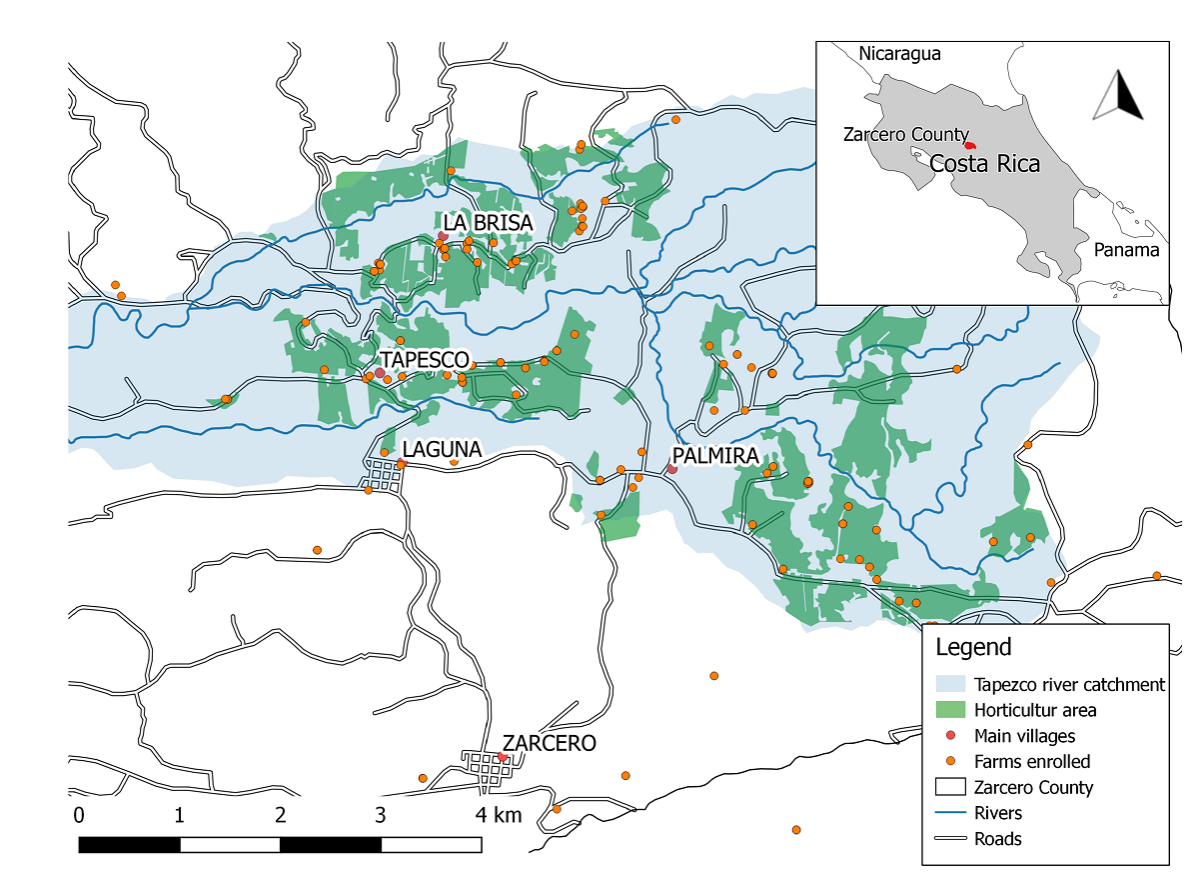
Study area (Tapezco river catchment) with global positioning system locations of 92 farms that were included in our study conducted in Zarcero County, Costa Rica, 2016. Shape files provided by Moraga (2015).

The study was conducted in the Tapezco river catchment in the Zarcero County, Costa Rica (Figure 3). This river catchment features approximately 760 small-scale smallholder farms with conventional and organic farming practices (~4 km^2^ of horticultural farms) [33] and has been previously used to monitor pesticide levels in the surface water near smallholder farms. Common crops in the area include potatoes, tomatoes, cabbage, carrots, lettuce, cilantro, and onions [32]; chlorothalonil, mancozeb, propineb, and phorate account for >50% of the pesticides used in the County. Notably, potatoes and onions are the crops with the highest pesticide use per hectare [32].

### Study Participants’ Selection and Study Visits

Conventional farms in the study area were identified using random global positioning system (GPS) points generated on the basis of smallholder land use data from 2015 (Moraga G, unpublished data, 2000) [34]. After a total of 200 GPS points were generated, study staff visited these locations and determined which ones corresponded to smallholder farms by contacting the farm owners or administrators. When the GPS point did not correspond to a horticultural farm, the closest smallholder farm within a radius of 1 km was registered; if no farm was nearby, the GPS point was dropped. Organic farms within the Tapezco river catchment or within 5 km from this area were identified using an existing list provided by the organic farmers’ association or through onsite identification.

After organic and conventional farms in the study area were identified, farm owners were briefly informed about the study aims and procedures (initial visits to the farms are, henceforth, called Information campaign; Figure 3). If they showed interest in participating in the study, basic contact information was collected to schedule a later visit to their farms to enroll study participants. Eligible participants were farm owners, permanent workers, or temporary pesticide applicators, all aged ≥18 years, who owned or worked in conventional farms located in the Tapezco river catchment or organic farms within or near the catchment area, and who did not have a diagnosis of psychiatric disease or used psychopharmacological medications.

Participants were visited twice during the study duration, either at the farms where they worked or at their homes (Figure 3). The baseline or initial study visits were conducted by two teams of 3 trained research assistants each and comprised 4 “*Stations* ” (duration ~45 minutes each) as follows: “*Station 1* ” included the administration of the informed consent and a structured questionnaire to collect data on sociodemographic characteristics, occupational history, pesticide exposure at work and at home, medical history including respiratory and allergic outcomes in the last 12 months, and a blood pressure measurement; “*Station 2* ” included the administration of a neurobehavioral test battery (eg, Purdue Pegboard and Finger Tapping Test) and a checklist of acute pesticide intoxication symptoms in the last 12 months; “*Station 3* ” included measurements of the erythrocytic AChE activity, anthropometry (ie, height, weight, and waist circumference), and blood pressure (second measurement) and urine sample collection; and “*Station 4* ” included the fNIRS assessment (only completed by a subset of study participants).

The follow-up study visits (duration ~15 minutes, 2-4 weeks after the first visit) were conducted by two trained research assistants and included the administration of a short questionnaire on recent pesticide exposure (administered to all participants) and farming practices (administered only to farm owners) and collection of toenail and hair samples and a second urine specimen.

All study instruments were pilot-tested in 10 farmers. Study protocols were administered by trained research assistants in Spanish, and data were entered directly into tablets (Samsung Galaxy Note 10.1 N8010) via an entry mask using Open Data Kit [35]. Questionnaires and other study instruments are available per request.

### Power and Sample Size Calculation

We based our sample size calculation on the difference in the erythrocytic AChE activity between organic and conventional farm owners and workers [36]. With a sample size of 300 farm owners and workers, the minimum number of farms to show a significant effect between the 2 groups was calculated to be 50 (ie, 25 conventional and 25 organic farms). In brief, we assumed an average cluster size of 6 farmers per individual farm (this assumption was derived from a pilot visit and expert opinion of local agronomists), an intraclass correlation coefficient (ICC) of 0.1, a ratio of SDs of 1.5 between exposed and unexposed persons regarding the erythrocytic AChE activity, a significance level of 5%, 80% power, and an effect size of 0.4, that is, a difference in the mean of the erythrocytic AChE activity between exposed and unexposed farm owners and workers of 0.4×√[(1 + 2.25)/2 SDs].

### Interviews and Self-Report Pesticide Exposure

Information on sociodemographic variables and occupational exposure to pesticides was collected using structured questionnaires (Table 1). During the baseline visit, study participants were asked about the crops that they had recently worked on and if they had prepared or applied pesticides. If they reported preparing or applying one of the 15 pesticides most commonly used in the study area (according to a previous study on good farming practices that was conducted in conventional farms from the study area, Table 2; [32]), detailed data on the mode of application, period, dose, frequency of pesticide applications, and personal protective equipment use were collected. In addition, participants were asked about their recent pesticide applications (prior to the collection of each urine sample), previous work in conventional or organic farms, and years of exposure to pesticides during their work life. During the follow-up visit, study participants were asked about changes in their work status and pesticide use since their baseline visit. In addition to the data described above, we collected information on farms’ characteristics (eg, size, type of crops grown on the farm, farming practices including pest control management, and water sources located nearby) using a structured questionnaire that was administered only to farm owners.

**Table 1:** Information collected during the baseline study visit using structured questionnaires.

Questionnaire section	Data collected
Sociodemographic characteristics	Age (years), sex, country of birth, years living in Costa Rica, years living in the Zarcero County, education level (last grade completed), handedness (right or left), smoking (number of cigarettes per day), alcohol consumption (number of glasses), family income (Costa Rican colones per household), computer literacy
Housing characteristics	Years living in the current house, number of bedrooms, number of people living in the house, type of water source, pet ownership (number and type), and farm animals living in or next to the house (number and type)
Work history	Age when started working in agriculture (years), age at first contact with pesticides (years), past jobs, and current jobs in agriculture
Pesticide use	Pesticide use (Table 2) during the last 12 months and last week, mode of application, frequency of pesticide applications (number of times), use of personal protective equipment (type and frequency), and personal hygiene habits (frequency)
Residential pesticide use	Indoor or outdoor pesticide use in the home (type)
Medical history	Respiratory and allergic symptoms, acute pesticide intoxications during the last 12 months (number of times), and other illnesses (type)

**Table 2:** Most frequently used pesticides (active ingredients; ordered from most frequently use to the least frequently used) in agricultural farms in the Zarcero County, Costa Rica^a^.

Active ingredient	Commercial names	Chemical subgroup	Group of action
Chlorothalonil	Bravo, Bravonil, Knight, Talonil, Thalonex, Folio Gold, Odeon	Chloronitriles	Fungicide
Benfuracarb	Oncol	Carbamates	Insecticide
Mancozeb	Dithane, Mancol, Ridomil, Titan	Dithiocarbamates	Fungicide
Boscalid	Bellis, Endura	Pyridinecarboxamids	Fungicide
Carbendazim	Afin, Cozaid, Crotonox, Carbendazina	Benzimidazoles	Fungicide
Acephate	Acefate, Orthene, Yucal	Organophosphates	Insecticide
Phorate	Forato, Thimet, Thimetoato, Timefor	Organophosphates	Insecticide
Fenamiphos	Fenemiphos, Nemacur	Organophosphates	Insecticide
Chlorpyrifos	Agromil, Batazo, Baygon, Lorsban, Solver, Terminator, Swat	Organophosphates	Insecticide
Glyphosate	Atila, Evigras, Ranger, Round Up	Glycine derivatives	Herbicide
Carbofuran	Carbodan, Curator, Furadan	Carbamates	Insecticide
Cypermethrin	Best, Cascabel, Cipermetrina, Combat, Cruz Verde, Tigre, Excalibur	Pyrethroids	Insecticide
Propamocarb	Acrobat CT, Previcur, Proplant, Prevalor	Carbamates	Fungicide
Paraquat	Gramoxone, Preglone, Rafaga	Bipyridiliums	Herbicide
Propineb	Antracol, Inicol, Taifen	Dithiocarbamates	Fungicide

^a^Modified from: Ramírez et al, 2016 [32]. Pesticides are ordered from most frequently used to the least frequently used.

### Biological Sample Collection and Analyses

#### Urine Samples

Spot urine samples were collected during the baseline and follow-up visits in plastic containers of 100 mL **(**Vacuette, sterile**)**. Specimens were stored at 4°C until the end of the fieldwork day. Then, samples were aliquoted in 15-mL plastic test tubes (PerformRTM Centrifuge tubes, Labcon, sterile) and stored at −20°C until their shipment at 4°C to Lund University, Sweden, for analysis.

Urine samples will be analyzed for multiple pesticide metabolites including, but not limited to, ethylenethiourea (ETU, a metabolite of mancozeb); propylenethiourea (PTU, a metabolite of propineb); 3,5,6-trichloropyridinol (TCP, a metabolite of chlorpyrifos); 3-phenoxybenzoic acid (3-PBA, a metabolite of pyrethroids permethrin, cypermethrin, deltamethrin, and cyfluthrin); and hydroxy pyrimethanil (a metabolite of pyrimethanil). These pesticides and their metabolites were selected because they are among the most commonly used in Zarcero [32] and for which biomarkers of exposure are available. Briefly, urine specimens will be analyzed using tandem mass spectrometry and high-performance liquid chromatography [37,38]. The limit of detection (LOD) are as follows: ethylenethiourea, 0.08 ng/mL; propylenethiourea, 0.1 ng/mL; TCP, 0.05 ng/mL; 3-PBA, 0.03 ng/mL; and hydroxy pyrimethanil, 0.1 ng/mL. In all sample batches, chemical blanks and in-house quality control samples will be included to ensure the quality of all measurements; additionally, the analyses of TCP and 3-PBA are part of the round robin interlaboratory comparison program (University of Erlangen-Nuremberg, Germany) with results within the tolerance limits. Furthermore, urine samples will be analyzed for creatinine and specific gravity so that pesticide metabolite concentrations can be adjusted for differences in urinary dilution [39].

#### Hair Samples

Hair samples (~20-30 strands) were collected from the occipital region, within 2 mm from the scalp, using stainless-steel scissors during the follow-up study visit. The samples were then stored at room temperature in sterile plastic bags until their shipment to the Federal University of Bahia, Brazil.

Hair specimens will be analyzed for manganese (Mn), which is contained in ethylene bisdithiocarbamate fungicides, such as mancozeb. Briefly, the nearest centimeter scalp (proximal end) of hair will be sonicated for 20 min in 0.5% Triton, rinsed 5 times with ultrapure water, sonicated for 10 min in 1-N nitric acid, rinsed once with 1-N nitric acid, and then rinsed 5 times with ultrapure water [40]. Approximately 10 mg of hair will be digested with 2 mL of concentrated HNO_3_ spectroscopic-grade acid in a microwave digestion oven (Mars-Express6, CEM, USA). The digested material will be diluted to 10 mL with ultrapure water. Hair samples, certified reference material (Human hair, International Atomic Energy Agency 086), and reagent blanks will be analyzed using electrothermal atomic absorption spectrometry with Zeeman background correction [41]. The analytical LOD for hair Mn concentrations will be set at 0.05 µg/L.

#### Toenail Samples

Toenail samples were collected during the follow-up study visit. Participants were asked to cut their toenails with clean stainless-steel nail clippers and put them inside of a sterile plastic bag. Toenail specimens were stored at room temperature until their shipment to Federal University of Bahia, Brazil.

Toenail samples will be analyzed for Mn using the same procedure described above for hair samples. Briefly, nails will be washed in a Triton X-100 solution, put in acetone, repeatedly rinsed with ultrapure water, and then dried in an oven. Later, the dried nails will be digested with spectroscopic-grade acid in a microwave digestion oven (Mars-Express6, CEM), diluted to 10 mL with ultrapure water, and analyzed electrothermal atomic absorption spectrometry with Zeeman background correction [42,43]. All processed samples and reference material from the International Atomic Energy Agency (ie, 085) will be analyzed in duplicates. The analytical LOD for toenail Mn concentrations will be set at 0.05 µg/L.

### Assessment of Health Outcomes

#### Symptoms of Acute Pesticide Poisoning

Participants were administered a checklist of symptoms of acute organophosphate and carbamate poisoning (eg, excessive salivation, lacrimation, vomiting, and diarrhea) during the 12-month period before the baseline study visit. This checklist has been previously used in studies of Latin American farm workers [28,44].

#### Respiratory and Allergic Outcomes

A short version of the European Community Respiratory Health Study II questionnaire [45] was administered to study participants to identify respiratory symptoms (eg, wheezing, shortness of breath, coughing, and phlegm), respiratory diseases (eg, asthma and chronic bronchitis), allergic outcomes (eg, rhinitis and eczema), and common respiratory hazards such as smoking and pet ownership [46]. This questionnaire has been previously used in studies of Costa Rican populations [47-49].

#### Neurobehavioral Outcomes

Study participants were administered the following eight neurobehavioral tests: Animals and words with initial letter “F” (to assess semantic and phonemic verbal fluency) [50]; Digit Span (working memory) [51]; Digit Symbol (visual perception abilities) [51]; Digit Vigilance (sustained attention and psychomotor speed) [52]; Trails Making Test Part A (executive function) [53]; Finger Tapping (psychomotor speed and coordination) [53]; Purdue Pegboard (fine motor function) [54]; and Brief Symptom Inventory (behavioral disorders including somatization, obsessive-compulsion, depression, anxiety, hostility, and psychoticism) [55].

These tests were selected on the basis of previous studies of Latin American populations exposed to pesticides [28,29], administration time, and cultural sensitivity [56]. Neurobehavioral assessments were conducted by two trained psychometricians and supervised by a physician with extensive experience on neurobehavioral testing. Quality assurance measures included pilot testing and review of recorded assessments.

#### Brain Activity

We used fNIRS (NIRSport, NIRx Medical Technologies, Los Angeles, CA, USA) to assess the cortical function associated with pesticide exposure in a random subsample of 50 study participants; a detailed description of our study methods can be found elsewhere [57]. Specifically, in this study, we hypothesized that higher pesticide exposures would be associated with more atypical brain activation patterns related to attention, working memory, and executive function. As these cognitive processes commonly elicit cortical activity within the bilateral dorsolateral prefrontal cortices [58-60], we targeted these regions with fNIRS. To engage our participants in tasks that required these cognitive abilities, each participant completed 3 computer-based tests that were optimized for neuroimaging applications. These tasks included the Wisconsin Card Sort test (an executive function and cognitive flexibility task) [60], Sternberg test (a letter-retrieval working memory task) [59], and Go/No-go test (an attention and impulse control task) [58]. Each task was conducted on a laptop computer that was dedicated to the fNIRS assessment and was completed on site.

#### Cardiometabolic Outcomes: Blood Pressure and Anthropometric Measurements

Trained research assistants measured systolic and diastolic blood pressure at two different time points (ie, at the beginning of “*Station 1* ” and “*Station 3* ” and about 1.5 hours apart from each other) during the baseline study visit using an automatic sphygmomanometer (Advantage 6021N). In addition, they measured participants’ height (cm) and weight (kg) using a portable stadiometer and a digital scale (Tanita BC533, Arlington Heights, IL, USA). Waist circumference (cm) was measured using a tape measure Hoechstmass (Hoechstmass Balzer GmbH, Sulzbach, Germany).

#### Erythrocytic Acetylcholinesterase Activity

Capillary blood samples were collected at the baseline visit according to the manual of the Test-mate ChE Cholinesterase Test System (Model 400; EQM Research Inc, Cincinnati, OH, USA). Briefly, a small lancet (size 30) was used to collect a small sample of 10 µm from the tip of the index finger of each study participant and placed into a capillary tube. Blood samples were analyzed on site for the erythrocytic AChE activity and hemoglobin levels using the same collection instrument [61].

### Statistical Analyses

We will explore differences in self-reported pesticide exposures and health outcomes (ie, respiratory and allergic as well as neurobehavioral and cardiometabolic outcomes, brain activity, symptoms of acute pesticide poisoning, and erythrocytic AChE activity) in farm workers from conventional and organic farms. Cumulative lifetime pesticide exposure, exposure during the last 12 months, and during the last week will be estimated using exposure intensity scores derived from a semiquantitative exposure algorithm (based on self-reported data on pesticides, personal protective equipment use, and personal hygiene habits) [18]. Exposure intensity scores will be calculated for each chemical family (eg, organophosphates and carbamates) and active ingredient (eg, phorate and chlorpyrifos) and then validated against urinary pesticide metabolite concentrations and hair and toenail Mn concentrations using Spearman correlation coefficients and multivariate mixed-effects regression models [22]. We aim to use multiple imputation techniques to replace pesticide metabolite or Mn concentrations below the limit of detection [62]. In addition, we will fit mixed-effects models to examine the reproducibility of urinary pesticide concentrations by calculating ICCs (an ICC of <0.50 indicates poor reliability) [63]. If appropriate, we will then average urinary pesticide metabolite concentrations across the repeated samples collected for each study participant.

Associations of pesticide exposure with health outcomes of interest will be examined using both exposure scores and biomarkers of exposure. More specifically, we will fit linear or logistic (depending on the outcome) mixed-effects regression models (with the variable “participant” as random effect and other variables, such as outcome and covariates, as fixed effects) to explore the association of pesticide exposure with the following: (1) self-reported symptoms of acute pesticide poisoning in the last 12 months; (2) self-reported symptoms of respiratory and allergic symptoms and outcomes; (3) neurobehavioral outcomes and brain activity; (4) cardiometabolic effects (ie, obesity and hypertension); and (5) erythrocytic AChE activity. These mixed-effects models will allow us to take into account the correlation between and within farms as well as between and within farm workers. Furthermore, we will fit generalized additive models to examine the nonlinearity of the exposure-outcome associations.

We will identify potential confounders and known predictors of the health outcomes of interest (eg, age and education level for neurobehavioral outcomes) using directed acyclic graphs and will include them *a priori* in our regression models. Furthermore, we will assess other potential confounders by adding them, one at a time, to the final models (models with *a priori* covariates). Additional covariates will be possibly included in the final models if they materially changed the magnitude of one or more exposure coefficients (>10%). Missing values (<10%) for covariates will be imputed by randomly selecting a value from the dataset or using multiple imputation techniques [62]. Statistical analyzes will be performed using STATA (Stata Corporation) and R (R Foundation for Statistical Computing).

## Results

The fieldwork for this study was conducted between May 2016 and August 2016. A total of 300 participants, including 113 farm owners and 187 workers from nine organic (48 participants) and 83 conventional (252 participants) horticultural smallholder farms from Zarcero County, Costa Rica, were enrolled. We had a 6.3% (281/300) loss to follow-up of study participants between the baseline and follow-up study visits (conducted ~2-4 weeks apart). During the study implementation, we observed that farms were, on average, smaller than we expected (about three owners or workers per farm) and that there were not as many organic farms in the study area as anticipated (only 10 farms out of the 25 that we expected). Interviews to farm owners and key community actors conducted during this study revealed that many organic farm owners had recently started using synthetic pesticides due to the increasing costs of growing organic produce and getting certified as organic producers. Given the limited number of organic farms in the study area, we decided to include all farm owners and workers from organic farms located in the study area or within 5 km from it. In addition, to reach the targeted sample of 300 participants, we had to enroll more conventional farm owners and workers than what we had anticipated. Data analyses are ongoing and expected to be published between 2019 and 2021.

## Discussion

This study has several limitations. First, its cross-sectional design will prevent us from identifying causal associations of pesticide exposure with health effects of interest. Second, we will not be able to exclude the possibility that there is recall or information bias, especially when relying on self-reported exposure to pesticides. In our study population, this bias may have worsened under the following conditions: (1) most study participants had a relatively low educational level; (2) pesticide use varied by crop and season; and (3) some farm owners did not communicate the specific pesticides that were used in their farms by their employees. Therefore, the validation of self-reported pesticide exposure data against biomarkers of exposure is crucial. Third, given that we had to enroll more conventional farm owners and workers than what we expected, and this could potentially threaten the internal validity of our study, we will assess participants’ pesticide exposures using exposure intensity scores and biomarker concentrations in addition to their farming practices. We will also run analyses using only data from the conventional farms to evaluate the effects of the miscalculated sample size on our exposure-outcome associations. Fourth, we observed some significant differences between owners and workers from conventional and organic farms (eg, seasonal workers, differences in country of origin, and education level) that could potentially confound the exposure-outcome associations. Hence, it was important to also collect detailed information on confounding factors and predictors of the outcomes of interest.

The limitations of this study are offset by notable strengths, including (1) the quantification of pesticide metabolites and Mn concentrations in different biological matrices, which will allow us to validate the exposure information collected via questionnaires (or at least data from recent exposures given the relatively short half-lives of some of the biomarkers of exposure); (2) the comparison of workers and owners from conventional and organic farms using comprehensive questionnaires on occupational pesticide exposure; and (3) the assessment of health outcomes using internationally standardized tests that will allow for direct comparison of the results from this study to those from studies of other populations.

This study is one of the first studies to examine the health effects of exposure to a wide range of pesticides on Latin American workers from conventional and organic farms. In addition, this is one the first epidemiological studies to examine the association of pesticide exposure with brain cortical activity in farm workers. We expect that this study will provide critical data on how occupational exposure to common pesticides may affect farm owners’ and workers’ health. Finally, we hope that this study will allow us to identify strategies to reduce pesticide exposure in farm workers and will lay the groundwork for a future longitudinal study of health outcomes in farm owners and workers exposed to pesticides.

## Appendix

Multimedia Appendix 1 Peer-review reports.

## Abbreviations

3-PBA: 3-phenoxybenzoic acid
AChE: acetylcholinesterase
fNIRS: functional near-infrared spectroscopy
GPS: global positioning system
ICC: intraclass correlation coefficient
LOD: limit of detection
LMICs: low- and middle-income countries
Mn: manganese
TCP: 3,5,6-trichloropyridinol
