# Supplementary material for: Exposure to Pesticides and Health Effects on Farm Owners and Workers From Conventional and Organic Agricultural Farms in Costa Rica: Protocol for a Cross-Sectional Study
Source: JMIR Res Protoc. 2019 Jan 25;8(1):e10914. doi: 10.2196/10914 (PMC6367668; doi:10.2196/10914)
Supplement: Multimedia Appendix 1 [file resprot_v8i1e10914_app1.pdf]

Die Ethikkommission
Nordwest- und Zentralschweiz
hat die vorliegenden Akten

☒ zur Kenntnis genommen
☒ genehmigt

Datum / Unterschrift
08.02.2017 / [illegible]

Swiss TPH

Swiss Tropical and Public Health Institute
Schweizerisches Tropen- und Public Health-Institut
Institut Tropical et de Santé Publique Suisse

Associated Institute of the University of Basel

Medizin & Diagnostik
Nationales Zentrum für importierte Parasitosen
Tropenmedizinisches Ambulatorium

Prof. Dr. med Christoph Hatz
Leitender Arzt

christoph.hatz@unibas.ch
Telefon 061 284 8255
Fax 061 284 8183

Prof. Dr. med. André Perruchoud
President
Ethikkommission Nordwest- und Zentralschweiz
(EKNZ)

Basel, 30 January 2017

Amendment to the research project "UBE 2016-00771"

Dear Prof. Perruchoud,

It is our pleasure to submit to you an amendment to the research project entitled "**Occupational exposure to pesticides and their effects on the health of workers in conventional and organic farms in the area Zarcero**" (your reference number UBE 2016-00771), for evaluation and approval by EKNZ.

The initial research protocol has been evaluated and approved by the EKNZ the Comité Ético Científico Universidad Nacional (CECUNA) in Costa Rica. The board stated that there are no principal ethical concerns with the protocol for our study in Costa Rica (a middle-income country). In the meantime the field work component of this study has been conducted successfully and we are now analyzing the data.

We now would like to apply the same methodology the Wakiso district in Uganda (a low-income country) between March 2017 and December 2018. Therefore, we adapted the protocol and added information on the second study site, while leaving the methodology otherwise as it was. We added in the title the study site "*Wakiso in Uganda*". Moreover, we will conduct an extensive pilot testing of the tools (information material, questionnaire, study tools) and will adapted them afterwards in accordance with the new context. In parallel we also submit the protocol to the "Higher Degrees, Research and Ethics Committee of Makerere University School of Public Health" in Uganda to obtain local ethical approval.

In brief, our research in Uganda is mainly marked by cross-sectional surveys to examine if the occupational exposure to pesticides harms the health of the farm workers in conventional and organic horticultural farms. Therefore, we aim to enrol 150 agricultural workers from conventional farms and 150 agricultural workers from organic farms that are visited by the research group on two occasions.

At the first visit we will (i) administer a structured questionnaire containing questions about socio-demographics, occupational history etc.; (ii) conduct neurobehavioral tests (e.g. short inventory of symptoms) with a simultaneous evaluation of brain function via

Swiss TPH

near-infrared spectroscopy (NIRS; in a subsample of the participants); (iii) obtain anthropometric parameters and blood pressure; (iv) measure the activity of the enzyme acetylcholinesterase; and (v) collect urine for quantification of pesticide and manganese metabolites. During a second visit (~1 month after the collection of primary samples) we will collect repeated urine samples and test the activity of the enzyme acetylcholinesterase. In addition, we will collect toenail and hair samples and administer a short questionnaire about recent pesticide exposure.

The results of the study are returned to the participants through written and verbal communication. The general results are returned to the communities in the region and to other stakeholders through restitution workshops, scientific articles in international magazines, presentations in national and international congresses and mass media.

We feel that the above described amendment to the protocol is straightforward and the involved techniques and interventions are the same and pose minimal risks from an ethical point of view and belongs to the Risk Category A (lowest risk) as with the initial protocol.

We look forward to your critical review and thank you for your consideration. Please contact us anytime if you need further information.

Yours sincerely

Prof. Dr. Christoph Hatz Dr Mirko Winkler

SWISS TROPICAL & PUBLIC HEALTH INSTITUTE
SCHWEIZ. TROPEN- & PUBLIC HEALTH-INSTITUT
Swiss TPH
BASEL

Enclosures:

1. Cover letter
2. Amendment UG Protocol synopsis
3. Amendment UG Patients Information and Consent Form
4. Amendment UG Study protocol
5. Amendment UG Questionnaire for first
6. Amendment UG Questionnaire second visit
7. Amendment UG List of Investigators
8. CVs of, Dr. Mirko Winkler, Dr. Samuel Fuhrimann, Prof. Dr. Christoph Hatz, Prof. Dr. Jürg Utzinger Dr. Ana Maria Mora Mora, Fernando Ramirez Muñoz, Dr. Christian Lindh, Dr. Jose Antonio Menezes Filho, Randall Gutiérrez Vargas and Daniel Rojas Valverde, Prof. Dr. Charles Niwagaba, Philipp Staudacher, Tiziana Manfioletti, Aggrey Atuhaire
9. LoA between Swiss TPH and EAWAG
10. LoA between Swiss TPH and Universidad National
11. LoA between Swiss TPH and SNIS
12. Ethical approval from Costa Rica

CC:

- Prof. Dr. Jürg Utzinger, Professor and Director, Swiss Tropical and Public Health Institute
- Prof. Dr. Christian Burri, Head of the Department for Pharmaceutical Medicine, Swiss Tropical and Public Health Insitute
- Dr. Ana Maria Mora Mora, Universidad Nacional, Heredia, Costa Rica

Ethikkommission Nordwest- und Zentralschweiz / EKNZ

Präsident: Prof. A. P. Perruchoud

Herr
Dr. M. Winkler, PhD
Swiss Topical- and Public Health-
Institute
Socinstrasse 57
4002 Basel

Basel, 08. Februar 2017

R E C H N U N G: UBE 2016-00771

- **EKNZ Tarifcode 3.3.2**

Betr.: **EKNZ: BASEC UBE 2016-00771- Amendment**
Occupational exposure to pesticides and their effects on the health of workers in conventional and organic farms in the area Zarcero

Unkostenbeitrag an die Ethikkommission Nordwest- und Zentralschweiz / EKNZ **SFR** **300.-**

Wir bitten Sie, diesen Betrag innerhalb von 30 Tagen auf unser Konto der Basler Kantonalbank (40-000061-4) zu überweisen und die Studiennummer der Ethikkommission zu erwähnen:

Konto Nr. IBAN: CH68 0077 0253 6185 2200 1
Ethikkommission Nordwest- und Zentralschweiz, Hebelstrasse 53, CH-4056 Basel
SWIFT-Code: BKBBCHBB

Mit freundlichen Grüssen

I. Oberli
Geschäftsführerin der Ethikkommission
Nordwest- und Zentralschweiz / EKNZ
